# A Case of Systemic Lupus Erythematosus Presenting with an Acute Abdomen: Successful Treatment with Steroid

**DOI:** 10.1155/2014/318939

**Published:** 2014-12-14

**Authors:** Haruka Fukatsu, Seisuke Ota, Koichi Sugiyama, Akinori Kasahara, Tadashi Matsumura

**Affiliations:** ^1^Department of Internal Medicine, Himeji St. Mary's Hospital, 650 Nibuno, Himeji 670-0801, Japan; ^2^National Hospital Organization, Minami-Okayama Medical Center, 4066 Hayashima, Okayama 701 0304, Japan; ^3^Department of Medicine and Clinical Science, Okayama University Graduate School of Medicine, Dentistry and Pharmaceutical Sciences, Okayama, Japan

## Abstract

Abdominal pain continues to pose diagnostic challenges for emergency clinicians. A 56-year-old Japanese woman was referred to our hospital due to severe abdominal pain which presented as occasional epigastric pain five months before and intermittent abdominal pain. She had a past history of ileus twice, for both of which laparotomy was performed without an alimentary tract resection. The wall thickening with marked three-wall structure from terminal ileum to sigmoid colon was seen and bladder wall was irregularly thick and enhanced irregularly. Among the differential diagnosis of the acute abdomen, autoimmune diseases were suspected, especially lupus erythematosus and Henoch-Schönlein purpura. On the second day of admission, abdominal pain worsened. The results of examinations of antinuclear antibody, anti-double-stranded DNA antibody, ANCA, and the complements were not obtained at that time; however, we started 1-g steroid pulse treatment for three days with success. With the results obtained later, the patient was given a diagnosis of probable systemic lupus erythematosus (SLE). The present case shows that SLE can present with acute abdomen and should be included in the wide range of the differential diagnosis of acute abdomen.

## 1. Introduction

Abdominal pain continues to pose diagnostic challenges for clinicians who practice emergency medicine. In many cases, differential diagnosis ranges widely from benign to life-threatening conditions. Causes include medical, surgical, intra-abdominal, and extra-abdominal ailments. Associated symptoms often lack specificity, and atypical presentations of common conditions are frequent, which complicate diagnosis and treatment further [1].

Collagen diseases cause abdominal pain; however, they are a relatively infrequent cause of acute abdomen compared with common gastrointestinal conditions, such as gastric ulcer, ileus, and colitis [2]. Here we report a patient with acute abdomen who was successively treated with prednisolone and ultimately diagnosed with systemic lupus erythematosus.

## 2. Case Presentation

A 56-year-old Japanese woman was referred to a clinician in April 2013 because of a 5-month history of occasional epigastric pain, 2-month history of intermittent abdominal pain/watery diarrhea, and a recent loss of appetite and worsening of watery diarrhea. Enhanced computed tomography (CT) revealed a thickening of the ascending colon and rectum. She was diagnosed with ileus and admitted to a local hospital. Fasting initially alleviated her symptoms. Eventually, she was discharged on the fifth day of hospitalization after she was able to eat without the recurrence of abdominal pain.

A week later, she experienced abdominal pain and watery diarrhea. Gastroenteroscopy and colonoscopy did not detect any abnormalities, and positron emission tomography/CT revealed no significant uptake of the tracer. Considering that a previous CT study had revealed the presence of gall bladder stones, cholecystectomy was planned after a provisional diagnosis of cholecystitis. On June 20th, her abdominal pain and watery diarrhea worsened, she was unable to eat, and she suffered from intermittent severe abdominal pain, predominantly in the upper abdomen; she was then referred to our hospital. She did not give history of travel abroad or consumption of uncooked meat, fish, or seafood in the recent past.

The patient gave a past history of two episodes of ileus, first in her twenties and then at 41 years of age. She had undergone laparotomy without an alimentary tract resection for both episodes. In addition, when she was in her thirties, she had been treated with a 10 mg/day dosage of prednisolone for 1 year because of the preliminary diagnosis of idiopathic thrombocytopenic purpura (ITP), on the basis of the decreased platelet counts. At the age of 54 years, she was referred to a rheumatologist because of elevated titers of autoantibodies against Ro/SSA and La/SSB and was diagnosed with Sjögren's syndrome. Serological testing at that time had revealed hypocomplementemia, but no other collagen diseases were detected; she was also diagnosed with hypertension. Her medications included amlodipine, lactomin, mosapride, sulpiride, and camostat mesilate. She had no history of allergy or family history of collagen diseases.

**Figure 1:**
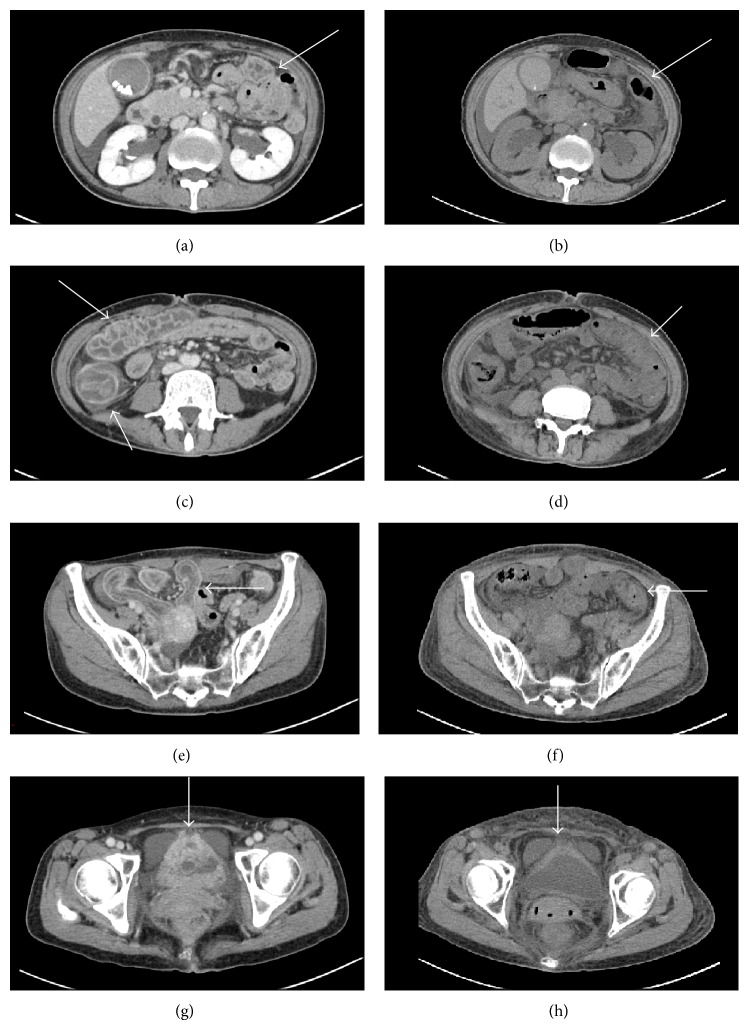
Enhanced abdominal CT on admission revealed an enlarged gall bladder (a, arrow), hydronephrosis, and ascites. The wall thickening with a marked three-wall structure extended from the terminal ileum to the sigmoid colon (c, e, arrows), and the bladder wall was irregularly thickened and enhanced (g, arrow). Eight days later, plain abdominal CT revealed an almost normal gall bladder wall (b, arrow), alimentary tract (d, f, arrow), and bladder (h, arrow).

Physical examination at admission revealed that she was alert but pale with a temperature of 37.1°C, blood pressure of 158/104 mmHg, pulse rate of 75 beats/min, respiratory rate of 20 breaths/min, and peripheral hemoglobin (Hb) oxygen saturation of 100%. Abdominal examination revealed abdominal fullness, weak bowel sounds, tenderness, but not rebound tenderness. Hematological findings were as follows: white blood cell (WBC) count: 1900/*μ*L with 87.5% neutrophils, red blood cell count: 3.98 × 10^6^/*μ*L, Hb: 11.4 g/dL, platelet count: 131.0 × 10^3^/*μ*L, and C-reactive protein concentration: 0.62 mg/dL. The results of tests for liver and renal function and electrolyte levels were normal. The serum amylase level was 37 IU/L, not elevated. Urinalysis or her electrocardiogram and chest X-ray revealed no abnormalities. Enhanced abdominal CT revealed gall bladder stones, enlarged gall bladder, hydronephrosis (Figure 1(a)), and ascites. In addition, wall thickening with marked three-wall structure extended from the terminal ileum to the sigmoid colon, and the bladder wall was irregularly thickened and enhanced (Figures 1(c), 1(e), and 1(g), arrow).

Thus, among the differential diagnoses of acute abdomen, autoimmune diseases were suspected, particularly lupus erythematosus and Henoch-Schönlein purpura. Her abdominal pain worsened on the second day of admission. The effect of pentazocine treatment was limited. The results of assays for antinuclear antibody, anti-double-stranded DNA antibody, antineutrophil cytoplasmic antibody, and complement levels were not yet available. However, we administered 1-g steroid pulse treatment for three days to avoid gastrointestinal perforation associated with both lupus erythematosus and Henoch-Schönlein purpura, considering that bacterial infection was clinically unlikely. Steroid treatment alleviated her abdominal pain and improved her appetite. The results of abdominal CT on the eighth day of admission showed a marked amelioration of gastrointestinal tract findings and bladder abnormalities (Figures 1(b), 1(d), 1(f), and 1(h)).

The results of her remaining blood tests, which became available later, were as follows: serum complement (CH50): 24.2 (26–48) units/mL; C3: 50 (86–160) mg/dL; and C4: 10 (17–45) mg/dL. Her autoantibody titers were as follows: antinuclear antibody: 1 : 1280 (normal, <40); anti-Sm antibody: 1 : 19.8 (normal < 10); anti-DNA antibody: 1 : 300 (normal < 6); anti-IgG antibody against single-stranded DNA: 1 : 565 (normal < 25); anti-IgG antibody against double-stranded DNA: 1 : 400 (normal, <12); anti-ribonucleoprotein antibody: 1 : 500 (normal, <10); anti-Ro/SSA antibody: 1 : 500 (normal, <10); and anti-La/SSB antibody: 1 : 109 (normal < 10). Thus, the patient fulfilled the following three criteria of systemic lupus erythematosus (SLE) of the American College of Rheumatology (1997): leukopenia < 4000/*μ*L (total) on at least two occasions, elevated titers of anti-DNA and anti-Sm antibodies, and elevated titers of antinuclear antibodies. Therefore, she was diagnosed with probable SLE.

She was discharged on the 30th hospital day with a daily oral 15 mg prednisolone. She was followed up in our outpatient clinic. Prednisolone was gradually decreased to 2.5 mg daily as she suffered from moon face in January 2014, 9 months after the admission. We added 25 mg azathioprine daily as WBC and platelet decreased in number. We increased azathioprine to 50 mg daily and prednisolone was discontinued in March. Follow-up examinations in April 2014 did not detect disease progression with 50 mg azathioprine daily.

## 3. Discussion

Systemic lupus erythematosus (SLE) is a chronic inflammatory disease of unknown cause that can affect the skin, joints, kidneys, lungs, nervous system, serous membranes, and/or other organs of the body. Immunologic abnormalities, especially the production of a number of antinuclear antibodies, are another prominent feature of the disease. Patients with SLE are subject to myriad symptoms, complaints, and inflammatory involvement that can affect virtually every organ. The most common pattern is a mixture of constitutional complaints with skin, musculoskeletal, mild hematologic, and serologic involvement [3, 4]. However, some patients have predominately hematologic, renal, or central nervous system manifestations. The pattern that dominates during the first few years of illness tends to prevail subsequently [5, 6].

The patient fulfilled three criteria of systemic lupus erythematosus (SLE) by the American College of Rheumatology in 1997 [7], namely, hematologic disorder of leukopenia less than 4000/*μ*L total on two or more occasions and of thrombocytopenia which is supported by the past diagnosis of ITP, immunologic disorders of an elevated titer of anti-DNA and anti-Sm antibody, and an abnormal titer of antinuclear antibody. Further, peritonitis causing ascites and hypocomplementemia supports a diagnosis of SLE. Successful treatment using prednisolone and azathioprine with a probable diagnosis of SLE suggests that the probable diagnosis was correct. In addition, the patient also meets the Systemic Lupus International Collaborating Clinics (SLICC) criteria [8]. Both the ACR and SLICC criteria are not diagnostic criteria; they are instead research-based classification criteria. Therefore, from a clinical standpoint, this patient has a diagnosis of systemic lupus.

Gastrointestinal (GI) manifestations occur in approximately 25%–40% of patients with SLE [9–12]. Many of these symptoms are nonspecific and often reflect either lupus of the GI tract or the effects of medication. Lupus mesenteric vasculitis (LMV) is a predominant cause of acute abdominal pain in SLE patients [13–15], and it occurs at a high frequency in association with active disease. LMV varies from 29% to 65% among SLE patients with acute abdominal pain. CT is the imaging method of choice to confirm the diagnosis because it permits the visualization of the bowel wall and abdominal vasculature. CT findings include characteristic dilated bowel loops, focal or diffuse bowel-wall thickening, abnormal bowel-wall enhancement (target sign), mesenteric edema, stenosis, or engorgement of the mesenteric vessels, causing the comb sign and ascites [16]. The CT findings in the present case were consistent with that of acute abdomen because of SLE.

Abdominal pain could be an initial presentation of SLE, including mesenteric vasculitis causing perforation, hemorrhage with peritonitis, acute pancreatitis, and intestinal pseudoobstruction, [16] but SLE could rarely present with acute abdomen. Abdominal pain of uncertain cause occasionally responds to steroids, suggesting an inflammatory mechanism [10, 12]; however, present case is clearly SLE.

We were able to avoid perforation in the present case by administering prednisolone before the serological test data were available. Although the decreased WBC count, normal C-reactive protein level (0.62 mg/dL), and imaging findings of enhanced abdominal CT were not consistent with a diagnosis of infectious colitis, the administration of steroid with limited data and a provisional diagnosis was a difficult choice, but we had a successful outcome.

## 4. Conclusions

We show here that SLE can present with acute abdomen and should be included in the wide range of differential diagnoses of acute abdomen. Therefore, we recommend that, in cases of acute abdomen with a preliminary diagnosis of SLE, clinicians should start treatment if the abdominal pain is severe, even before test results are available.
